# Prognostic Biomarkers in Isocitrate Dehydrogenase Wild-Type Glioblastoma: A Focus on B7-H3

**DOI:** 10.3390/brainsci15020212

**Published:** 2025-02-19

**Authors:** Ramazan Oğuz Yüceer, Seyhmus Kaya, Sema Nur Balcı, Hatice Reyhan Eğilmez, Mukaddes Yılmaz, Eda Erdıs

**Affiliations:** 1 Department of Pathology, Sivas Cumhuriyet University School of Medicine, 58140 Sivas, Turkey; drseyhmuskaya21@gmail.com (S.K.); egilmezreyhan@gmail.com (H.R.E.); 2 Sivas Cumhuriyet University School of Medicine, 58140 Sivas, Turkey; semanurbalci38@gmail.com; 3 Department of Clinical Oncology, Sivas Cumhuriyet University School of Medicine, 58140 Sivas, Turkey; ylmzmukaddes@gmail.com; 4 Department of Radiation Oncology, Sivas Cumhuriyet University School of Medicine, 58140 Sivas, Turkey; dr.erdiseda@gmail.com

**Keywords:** glioblastoma, B7-H3, PD-L1, immune checkpoint, survival, IDH wild type, brain tumor, biomarker, CD276, adult-type diffuse glioma

## Abstract

Background: Isocitrate dehydrogenase (IDH) wild-type (wt) glioblastoma is an aggressive malignancy associated with poor clinical outcomes, marked by high heterogeneity and resistance to treatment. This study aims to investigate the prognostic significance of B7-H3 expression in IDH wt glioblastoma and its potential association with clinical outcomes, including overall survival (OS) and progression-free survival (PFS). Additionally, the relationship between B7-H3 and PD-L1 expression was explored. Methods: A retrospective cohort of 86 IDH wt glioblastoma patients, all of whom underwent surgery, radiotherapy, and temozolomide treatment, was analyzed. B7-H3 expression was quantified using an immunoreactivity score (IRS), classifying samples as low (IRS ≤ 4) or high (IRS > 4). PD-L1 expression was evaluated based on tumor and immune cell staining, with >5% positivity indicating significant expression. Results: High B7-H3 expression was significantly associated with poorer OS and PFS. Co-expression of B7-H3 and PD-L1 was prevalent, particularly among younger male patients with unifocal tumors; however, PD-L1 expression did not show a significant correlation with clinical outcomes. Conclusions: B7-H3 appears to be a promising prognostic biomarker in IDH wt glioblastoma and may serve as a target for developing combination therapies, integrating B7-H3-targeting treatments with immune checkpoint inhibitors. Further prospective studies are necessary to validate these findings and to explore potential therapeutic strategies.

## 1. Introduction

Grade 4 isocitrate dehydrogenase (IDH) wild-type (wt) glioblastoma is the most aggressive and frequently occurring glioma in adults, comprising approximately 90% of adult-type diffuse gliomas according to the fifth edition of the World Health Organization (WHO) classification of tumors of the central nervous system (CNS) criteria, as published in 2021 [1]. Distinguished by its rapid proliferation and extensive infiltration into the surrounding brain tissue, grade 4 IDH wt glioblastoma is associated with poor clinical outcomes despite advancements in neurosurgical techniques, radiotherapy, and chemotherapy [2]. In contrast to IDH-mutant glioblastomas, which predominantly manifest in younger individuals, often develop as secondary brain tumors, and are moderately linked to improved survival outcomes, grade 4 IDH wt glioblastomas typically emerge de novo in older patients [3]. The IDH wt molecular subtype exhibits distinct genetic alterations, including epidermal growth factor receptor (EGFR) amplification, telomerase reverse transcriptase (TERT) promoter mutations, and homozygous deletion of cyclin-dependent kinase inhibitor 2A/B (CDKN2A/B) [4,5]. These genetic changes drive oncogenesis, enhance tumor aggressiveness, and contribute to therapeutic resistance. Additionally, the highly immunosuppressive tumor microenvironment and significant intratumoral heterogeneity further complicate effective management. Standard treatment protocols for grade 4 IDH wt glioblastoma consist of maximal safe resection followed by adjuvant radiotherapy with concurrent and adjuvant temozolomide [6]. Despite these interventions, prognosis remains poor, with a median overall survival (OS) of approximately 12 months and a 5-year survival rate of less than 10% [2]. The complex pathophysiology and therapeutic challenges associated with grade 4 IDH wt glioblastoma highlight the urgent need for innovative treatment strategies and robust prognostic biomarkers to improve clinical outcomes and enable personalized therapeutic interventions.

B7 homolog 3 (B7-H3) (CD276), a member of the B7 superfamily of immunoregulatory molecules, plays a pivotal role in tumor immunology and cancer progression [7,8]. Aberrant overexpression has been observed in various malignancies, including lung, breast, prostate, pancreatic, and CNS tumors [9,10,11,12,13,14,15,16,17]. Mechanistically, B7-H3 exerts dual immunomodulatory functions as an immune checkpoint molecule, suppressing T-cell-mediated anti-tumor responses while simultaneously facilitating tumor-promoting processes such as tumor cell invasion, angiogenesis, and resistance to treatment [7,8]. In glioblastoma, elevated B7-H3 expression is closely linked to aggressive clinicopathological characteristics, including a higher tumor grade, increased invasiveness, and poorer OS outcomes [16,17]. Furthermore, B7-H3 contributes to immune evasion by inhibiting natural killer (NK) cell activity and fostering an immunosuppressive tumor microenvironment [18]. Preclinical studies have demonstrated the potential therapeutic efficacy of B7-H3-specific monoclonal antibodies and antibody–drug conjugates (ADCs) in glioblastoma models [19]. These findings highlight the prognostic and therapeutic significance of B7-H3 in glioblastoma, positioning it as a crucial target in the rapidly advancing field of cancer immunotherapy.

Programmed cell death ligand-1 (PD-L1) has emerged as a central focus in cancer immunotherapy owing to its critical role in immune evasion and tumor progression. In glioblastoma, elevated PD-L1 expression is associated with poor prognosis, reduced OS, and increased resistance to conventional treatments, such as chemotherapy and radiation [20,21]. Glioblastomas, which are characterized by their aggressive nature and resistance to standard therapies, have shown promising potential as immune checkpoint inhibitors targeting the PD-1/PD-L1 axis in clinical trials. For instance, in the Keynote-028 study, pembrolizumab monotherapy was administered to 26 patients with recurrent glioblastoma; however, no significant responses were observed in the majority of patients [22]. Similarly, the Checkmate-143 trial revealed that nivolumab did not significantly prolong OS compared with bevacizumab [23]. These findings suggest that factors such as the immunosuppressive properties of glioblastoma and the challenges posed by the blood–brain barrier may limit the efficacy of PD-L1 inhibitors. Despite these challenges, the clinical efficacy of PD-L1-targeted therapies remains variable, and ongoing research has focused on identifying biomarkers that can predict the response to such treatments. Overall, while PD-L1 has substantial prognostic and therapeutic potential, further studies are necessary to optimize its application in glioblastoma and address resistance mechanisms.

Recent studies have explored the efficacy of bispecific ADCs targeting B7-H3 and PD-L1. For instance, one study found that BsADCs outperformed monotherapy, particularly in terms of tumor cytotoxicity both in vitro and in vivo, and enhanced the cytotoxic activity of immune cells against tumor cells [24]. Additionally, bispecific ADCs bolster tumor-specific immune responses by inducing immunogenic cell death (ICD) and endoplasmic reticulum stress [24]. Similarly, the combination of enoblituzumab, an anti-B7-H3 monoclonal antibody, and pembrolizumab (anti-PD-1) demonstrated an acceptable safety profile and anti-tumor activity in immune checkpoint inhibitor-naive patients [25]. This combination therapy is associated with increased objective response rates in patients with head and neck squamous cell carcinoma (HNSCC) and advanced-stage non-small cell lung cancer (NSCLC) [25]. These findings suggest that B7-H3 and PD-L1-targeted ADCs, when combined with immune checkpoint inhibitors, may offer promising synergistic potential in cancer treatment.

This study aimed to explore the prognostic impact of co-expression of B7-H3 and PD-L1 in tumor tissue on clinical outcomes, treatment efficacy, and OS in grade 4 IDH wt glioblastoma, a subgroup characterized by high recurrence rates and poor response to treatment. Furthermore, it offers an innovative perspective on the design of therapeutic strategies targeting key points in the immune checkpoint pathway as well as ADC combination therapies, which may inform future clinical studies on the management of CNS tumors.

## 2. Materials and Methods

### 2.1. Randomization of Participants, Data Collection, and Clinical Follow-Up

**Figure 1:**
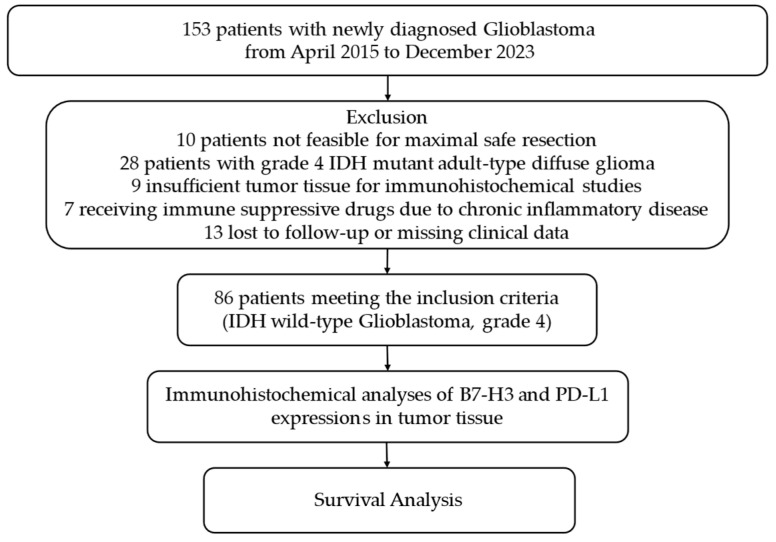
Flowchart of this study according to CONSORT diagram. Abbreviations: PD-L1, programmed cell death ligand 1; IDH, isocitrate dehydrogenase; CONSORT, consolidated standards of reporting trials.

This retrospective study examined data from 153 patients diagnosed with grade 4 adult-type diffuse glioma, which was pathologically confirmed based on the 2021 WHO classification. The patients were treated at Sivas Cumhuriyet University School of Medicine Research and Practice Hospital between April 2015 and December 2023. The exclusion criteria included 10 patients who did not undergo surgical resection, 28 patients with grade 4 IDH-mutant astrocytoma representing a subgroup with distinct biological characteristics, 9 patients with insufficient tumor tissue for immunohistochemical analysis, and those on immunosuppressive medications for chronic inflammatory diseases. Additionally, 13 patients were excluded due to loss to follow-up or inaccessible data. The final cohort included 86 patients with complete clinical, radiological, and laboratory records, as shown in the flowchart (Figure 1).

The collected data included demographic and clinical variables such as age, sex, Eastern Cooperative Oncology Group Performance Status (ECOG PS), comorbidities, tumor characteristics (lateralization, origin lobe, focality, and tumor size), surgical approach, Ki67 proliferation index, p53 mutational status, presence of alpha thalassemia/mental retardation syndrome X-linked (ATRX) loss, IDH mutation status, adjuvant therapies, therapeutic response, disease progression, subsequent treatment strategies, and OS.

All patients received standard treatment according to the National Comprehensive Cancer Network (NCCN) guidelines, which included concurrent radiotherapy and temozolomide, followed by adjuvant temozolomide. Throughout the sequential treatment process, encompassing surgery and radiotherapy, all patients underwent regular clinical monitoring, which included periodic assessments using serial MRI scans. Clinical responses were classified according to the Revised Response Evaluation Criteria in Solid Tumors (RECIST) version 1.1. Progression-free survival (PFS) was defined as the time from the date of pathological diagnosis to the occurrence of disease progression, death, or the last follow-up. OS was defined as the duration from the date of pathological diagnosis to death from any cause or last follow-up.

This study was conducted in accordance with the Declaration of Helsinki, as adopted in 1964 and revised in 2013, with strict adherence to the ethical principles. The study protocol was comprehensively reviewed and approved by the Non-Interventional Clinical Research Ethics Committee of Sivas Cumhuriyet University on 16 May 2024 (Approval Number: 2024/05-41). Due to the retrospective nature of this study, patient consent was not required. However, all data were anonymized to ensure patient confidentiality.

### 2.2. Immunohistochemical Evaluation of B7-H3 and PD-L1 Expression in Tumor Tissues

Hematoxylin and eosin (H&E)-stained tumor sections from patients diagnosed with grade 4 IDH wt glioblastoma were re-evaluated, and paraffin blocks containing tumor tissue with normal glial components and no necrosis were selected. From these blocks, 3–4 µm thick sections were prepared and mounted onto adhesive-coated slides. The sections were incubated with primary antibodies against CD276 (B7-H3) (rabbit monoclonal antibody, clone EPR20115, 1:4000 dilution, Abcam, Cambridge, UK) and PD-L1 (rabbit monoclonal antibody, SP142, 1:100 dilution, Roche Ventana, Oro Valley, AZ, USA). Positive control tissues (tonsil tissue for B7-H3 and PD-L1) were processed alongside the tumor samples for validation. Immunohistochemical staining was performed using a fully automated system (Roche Ventana Benchmark Ultra, Basel, Switzerland).

**Figure 2:**
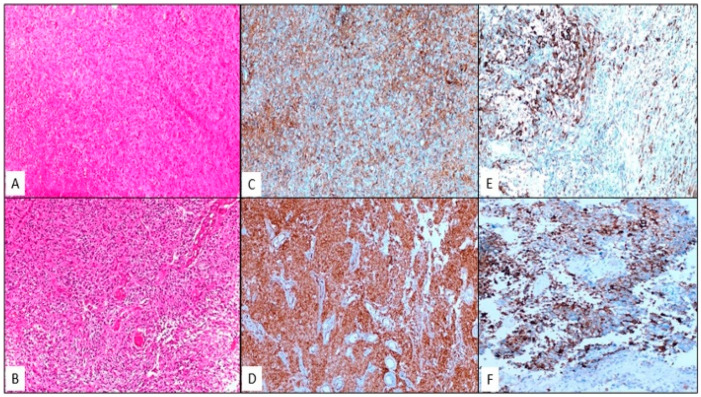
(**A**,**B**) IDH wild-type glioblastoma demonstrating increased cellularity, prominent pleomorphic tumor cells, abundant vascular proliferation, and focal geographic necrosis (H&E, ×200). (**C**) Low expression of B7-H3 in IDH wild-type glioblastoma. (**D**) High expression of B7-H3 in IDH wild-type glioblastoma. (**E**) Tumor cells with 15% positive staining for PD-L1. (**F**) Tumor cells with 80% positive staining for PD-L1 (DAB, ×200).

Immunohistochemically stained slides were independently evaluated by three pathologists (R.O.Y., H.R.E., and S.K.) with expertise in neuropathology, who were blinded to clinical details. The evaluation was performed using a validated semi-quantitative method to calculate the immunoreactivity score (IRS). For B7-H3, positive membranous and/or cytoplasmic staining was considered. For B7-H3, positive membranous and/or cytoplasmic staining was assessed. The staining intensity was graded as follows: 0, none; 1, weak; 2, moderate; and 3, strong. The extent of staining in the tumor cells was scored as 0 = 0%, 1 = 1–19%, 2 = 20–50%, and 3 = >50%. IRS was calculated as the product of the staining intensity and the proportion of positive cells, with samples categorized as low (IRS < 4) or high (IRS ≥ 4) [15]. For PD-L1, cytoplasmic staining of the tumor and immune cells was evaluated. Tumor samples with <5% staining were classified as negative, whereas those with >5% staining were classified as positive [21]. Representative sections of immunohistochemical evaluations are shown in Figure 2.

### 2.3. Statistical Analysis

Statistical analyses were conducted using Statistical Package for Social Sciences (SPSS) version 27 for Windows (IBM SPSS Inc., Chicago, IL, USA). The required sample size was determined using G-Power 3.1.9.7. Assuming an effect size of 0.50, a type I error rate of 0.05, and a power of 95%, it was calculated that 75 tissue samples would be sufficient for this study. The normality of the data distribution was assessed using the Kolmogorov–Smirnov test. Numerical variables with a normal distribution are presented as mean ± standard deviation, whereas those with a non-normal distribution are expressed as median (min–max). The relationship between B7-H3 and PD-L1 expression levels and clinical or pathological markers was evaluated using the chi-squared test or Fisher’s exact test. Spearman’s correlation coefficients were calculated to compare frequency distributions between the tested groups. OS and PFS were estimated using the Kaplan–Meier method and compared using the log-rank test. The relationships between variables and survival outcomes were assessed using univariate Cox regression analyses, with significant variables subsequently included in a multivariate Cox regression model. The Bonferroni correction was applied to control the Type I error rate (false-positive results) in the context of multiple comparisons. Statistical significance was defined as *p* < 0.05 for all analyses.

## 3. Results

The median age of the study cohort was 59 years (range: 25–84), with 50% of the patients aged 60 years or older. Forty-nine patients (57.0%) were male. Comorbidities were present in 44 patients (51.2%), with hypertension being the most common (62.1%). Right hemisphere involvement was more frequent than left, with the temporal lobe being the most commonly affected site (36.0%), while the occipital lobe was the least affected (17.4%). The ECOG PS was 0–1 in 42 patients and ≥2 in 44 patients. Single-focal tumors were observed in 62 patients (72.1%), and subtotal resection was performed in 54.7% of cases. Sixty-three patients (73%) completed adjuvant RT. The median tumor size on MRI was 50 mm (range, 10–90 mm), with a tumor size exceeding 50 mm in 48.8% of cases. ATRX loss was detected in 10 patients (11.6%), the Ki67 proliferation index was greater than 50% in 27 patients (31.4%), and p53 mutation was identified in 45 patients (52.3%). The clinical and demographic characteristics of the patients are summarized in Table 1.

B7-H3 expression was negative/low in 39 patients (45.5%) and high in 51 patients (54.7%) in the grade 4 IDH wt glioblastoma cohort. High B7-H3 expression was more frequently associated with younger age, male sex, absence of comorbidities, lower ECOG PS, right and temporal lobe localization, receipt of adjuvant chemotherapy, gross total resection, absence of ATRX loss, low Ki67 levels, and presence of p53 mutations. However, no statistically significant differences were observed (*p* > 0.05). A significant relationship was found between B7-H3 expression and patients who received radiotherapy as well as those with a single-focus tumor (*p* < 0.05) (Table 1).

PD-L1 expression was negative in 67 (77.9%) and positive in 19 (22.1%) grade 4 IDH wt glioblastoma patients. Although PD-L1 expression was more frequently observed in younger patients, males, those without comorbidities, with low ECOG PS, right and temporal lobe involvement, those receiving chemotherapy and radiotherapy, with single-focus tumors, gross resection, absence of ATRX loss, low Ki67 levels, and presence of p53 mutation, no statistically significant differences were found (*p* > 0.05) (Table 1).

Although PD-L1 positivity was more frequent in patients with high B7-H3 expression, no significant correlation was observed (*p* = 0.137, r = 0.147). Normal glial tissue adjacent to the tumor was observed in 50 patients. No expression of B7-H3 or PD-L1 was observed in normal glial tissues, which was statistically significant compared to that in the tumor tissue (*p* < 0.001).

### Survival Analysis

**Figure 3:**
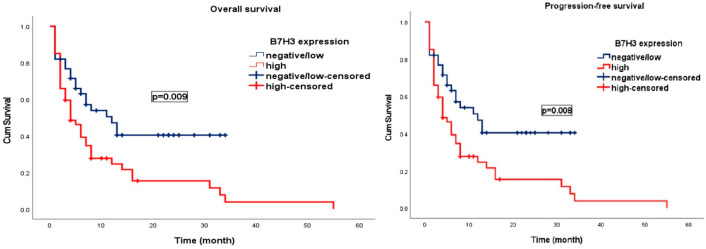
Kaplan–Meier curves illustrating the survival outcomes of patients with IDH wild-type glioblastoma, stratified by B7-H3 expression levels.

**Figure 4:**
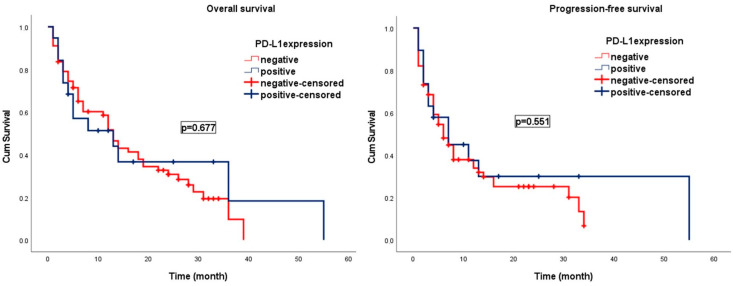
Kaplan–Meier curves illustrating the survival outcomes of patients with IDH wild-type glioblastoma, stratified by PD-L1 expression levels.

During a median follow-up period of 15.4 months (95% CI; 1.0–49.6 months), progression was observed in 74 patients (86.1%) and 62 patients (72.1%) died. In patients with grade 4 IDH wt glioblastoma, the median OS and PFS were 13.0 ± 1.11 months (95% CI; 10.8–15.2) and 7.0 ± 1.1 months (95% CI; 4.9–9.1). In patients with negative/low B7H3 expression, the median OS was 22.0 months (95% CI; 7.2–36.8) and PFS was 12.0 months (95% CI; 6.0–18.0), whereas in those with high B7H3 expression, the median OS was 8.0 months (95% CI; 3.2–12.8) and PFS was 4.0 months (95% CI; 1.9–6.1). Higher B7H3 expression was associated with significantly poorer OS and PFS (*p* = 0.009 and *p* = 0.008, respectively) (Figure 3 and Figure 4).

Among patients with PD-L1-negative expression, the median OS was 13.0 months (95% CI; 10.6–15.4), similar to the 13.0 months (95% CI; 1.0–27.6) observed in PD-L1-positive patients. The median PFS was 6.0 months (95% CI; 3.5–8.5) in the PD-L1-negative patients and 7.0 months (95% CI; 1.0–15.0) in the PD-L1-positive patients. No statistically significant association was identified between PD-L1 expression levels and either OS or PFS (*p* = 0.677 and *p* = 0.551, respectively) (Figure 3 and Figure 4).

In the univariate analysis conducted using the Cox proportional hazards model, advanced age, presence of comorbidities, high ECOG PS, temozolomide treatment, and elevated B7H3 expression were identified as significant predictors of OS (*p* < 0.05) (Table 2). Multivariate analysis revealed that adjuvant temozolomide and high B7H3 expression were independently associated with OS (*p* < 0.05) (Table 3).

In the univariate Cox proportional hazards model assessing potential risk factors for PFS, advanced age and elevated B7H3 expression were significantly associated with a shorter PFS (*p*< 0.05) (Table 2). Similarly, in multivariate analysis, advanced age and high B7H3 expression remained independently and significantly associated with PFS (*p* < 0.05) (Table 3).

## 4. Discussion

Grade 4 IDH wt glioblastoma is characterized by its highly heterogeneous biology, frequent recurrence, and aggressive clinical course, leading to elevated mortality rates despite advances in treatment approaches [3]. In recent years, reflecting trends across oncology, clinical research on immunotherapy and targeted therapies has gained increasing prominence in managing this malignancy. ADC-targeting pathways, such as BRAF/MEK, NTRK, and mTOR, have been integrated into the therapeutic arsenal for refractory cases, demonstrating notable clinical efficacy [26,27,28,29]. Similarly, immune checkpoint inhibitors, including nivolumab and pembrolizumab, which target PD-1 and PD-L1, have been evaluated for recurrent glioblastoma [22,23]. However, their response rates fell short of expectations. Critical challenges, such as insufficient penetration of the blood–brain barrier and unresolved concerns regarding intracranial efficacy, remain key areas requiring further investigation.

Glial tumors are immunologically categorized as “cold” tumors, characterized by low infiltration of immune cells within their microenvironment, which limits the activation of immune responses essential for immunotherapy efficacy [30]. In addition, these tumors actively create an immunosuppressive environment that enhances their capacity to evade immune surveillance. Immunosuppression is mediated through the recruitment of regulatory T cells (Tregs) and macrophages with an M2 phenotype, both of which inhibit antitumor immunity. High PD-L1 expression in tumor cells further reduces immune recognition by impairing the activation of cytotoxic T cells [20]. The genetic and molecular heterogeneity of brain tumors also contribute to immunotherapy resistance by promoting diverse mechanisms of immune evasion [31]. Moreover, glioblastomas, such as malignant brain tumors, typically exhibit a low tumor mutation burden, which limits the generation of neoantigens needed to stimulate robust immune responses [32]. Given these challenges, strategies to improve immunotherapy efficacy in brain tumors focus on developing combination therapies and innovative approaches aimed at modulating the immunosuppressive microenvironment and overcoming the blood–brain barrier.

B7-H3 is a transmembrane immune regulatory protein expressed in a variety of solid tumors, including glioblastoma. Its overexpression is associated with enhanced tumor proliferation, invasion, and neovascularization, establishing it as an unfavorable prognostic biomarker linked to poorer clinical outcomes in glioblastoma [17]. In glioblastoma samples exhibiting high B7-H3 expression, it has been reported that this molecule enhances the immune-suppressive capabilities of macrophages by promoting their polarization towards the M2 phenotype, thereby facilitating tumor growth [8]. Research has suggested that this interaction between B7-H3 and the M2 phenotype may be linked to poor prognosis in patients with aggressive tumors such as glioblastoma. This relationship highlights the potential necessity of targeting B7-H3 in immunotherapies and suggests the potential benefits of therapeutic strategies aimed at modulating the M2 phenotype [8]. In addition to its prognostic significance, preclinical studies have demonstrated the antitumor efficacy of B7-H3-targeting ADCs in glial tumor models [19]. Notably, a recent study by Johnson and colleagues reported promising outcomes with ifinatamab deruxtecan, a novel ADC combining B7-H3 targeting and topoisomerase inhibition, achieving objective response rates of up to 52% in small cell lung cancer (SCLC) [33]. Although immune checkpoint inhibitors targeting PD-L1 and PD-1 have exhibited limited efficacy as monotherapy for glioblastoma, emerging evidence from bi-specific ADC studies incorporating B7-H3 with either PD-L1 or PD-1 has shown encouraging antitumor activity, highlighting the potential of these combination approaches in addressing therapeutic resistance [24,25].

The findings of this study substantiate the association between elevated B7-H3 expression in tumor tissue and significantly poorer OS and PFS in patients with grade 4 IDH wt glioblastoma. Furthermore, comprehensive regression analyses highlighted the strong predictive potential of B7-H3 expression for clinical outcomes. A recent experimental study on glioblastoma identified a significant correlation between B7-H3 expression and increased resistance to temozolomide therapy, as well as elevated recurrence rates in in vitro models [34]. Given its role in tumor immune evasion, B7-H3 has been recognized as a promising target for the development of combination-based immunotherapeutic strategies, particularly in refractory glioblastoma [35]. The clinical data from this study, which focus on a distinct subgroup specifically characterized by grade 4 IDH wild-type glioblastoma, further reinforce the therapeutic potential of B7-H3, as highlighted in the existing literature. The lack of a significant relationship between PD-L1 expression and glioblastoma clinical data may be attributed to the fact that patients exhibiting positive PD-L1 expression comprised a very small proportion of the cohort. Nonetheless, the frequent observation of high coexpression of B7-H3 and PD-L1, particularly in younger male patients without comorbidities and those with unifocal tumors, underscores the potential interactions between the tumor microenvironment (TME) and immune checkpoint pathways. However, to gain a deeper understanding of the functional effects of B7-H3 on the tumor immune microenvironment, further studies utilizing in vitro and in vivo models are necessary.

Given the growing recognition of the potential of combination therapies in the development of new treatment strategies for such aggressive tumors, clinical trials targeting B7-H3-specific ADC combinations with both anti-VEGF and anti-PD-1/PD-L1 therapies may present a promising approach for optimizing therapeutic regimens. In conclusion, this study offers a novel perspective on tumor immunology, providing insights into promising strategies for future clinical research aimed at broadening treatment options, overcoming resistance mechanisms, and enhancing clinical outcomes.

This study, which stands out with its innovative hypothesis, focuses on grade 4 IDH wt glioblastoma, a cancer that represents an area where clinicians face significant challenges owing to its aggressive biological behavior and extremely limited treatment options. However, this study has several limitations. First, factors such as its retrospective design, reliance on single-center data, and narrow cohort may negatively affect the strength of this study. The lack of an internationally recognized scoring system for interpreting B7-H3 expression in tumor tissues through immunohistochemical analysis and the absence of an independent validation cohort may also diminish the reliability of this study. Additionally, variations in the treatment options selected by clinicians in advanced-line therapy could contribute to the potential misinterpretation of OS outcomes. Furthermore, the inability to systematically investigate pathological evaluations that may influence prognosis, such as O6-methylguanine-DNA-methyltransferase (MGMT) methylation, TERT promoter mutations, EGFR amplification, and CDKN2A/B deletions due to cost-effectiveness or technical limitations, may also constrain the interpretation of this study’s results.

## 5. Conclusions

Based on the findings of this study, it can be concluded that high B7-H3 expression in grade 4 IDH wt glioblastoma is significantly associated with poorer OS and PFS. The identification of B7-H3 as a negative prognostic biomarker highlights its potential as a target for future therapeutic strategies, particularly combination therapies. Despite the challenges of immunotherapy in glioblastoma, the promising outcomes of bispecific ADCs targeting both B7-H3 and immune checkpoints, such as PD-L1 or PD-1, open new possibilities for overcoming resistance mechanisms. While this study provides valuable insights, its retrospective design, small cohort size, and lack of internationally standardized immunohistochemical scoring systems for B7-H3 expression should be acknowledged as limitations. Future clinical trials combining B7-H3-targeted therapies with anti-VEGF and anti-PD-1/PD-L1 agents may offer significant potential for improving the treatment outcomes in glioblastoma.

## Figures and Tables

**Table 1:** The classification of sociodemographic and clinicopathological data according to the B7-H3 and PD-L1 expression levels of the tumor tissue in patients with grade 4 IDH wt glioblastoma (all patients, *n* = 86).

Variables, *n* (%)			B7-H3 Expression			PD-L1 Expression		
			Negative/Low	High	*p*	Negative	Positive	*p*
			*n*, (%)	*n*, (%)		*n*, (%)	*n*, (%)	
Age	<60	43 (50.0)	19 (48.7)	24 (51.1)	0.500	33 (49.3)	10 (52.6)	0.500
	≥60	43 (50.0)	20 (51.3)	23 (48.9)		34 (50.7)	9 (47.4)	
Sex	Female	37 (43.0)	19 (48.7)	18 (38.3)	0.226	29 (43.3)	8 (42.1)	0.570
	Male	49 (57.0)	20 (51.3)	29 (61.7)		38 (56.7)	11 (57.9)	
Comorbidity	None	42 (48.8)	17 (43.6)	25 (53.2)	0.252	32 (47.8)	10 (52.6)	0.454
	Present	44 (51.2)	22 (56.4)	22 (46.8)		35 (52.2)	9 (47.4)	
ECOG PS	0–1	42 (48.8)	17 (43.6)	25 (53.2)	0.252	32 (47.8)	10 (52.6)	0.454
	≥2	44 (51.2)	22 (56.4)	22 (46.8)		35 (52.2)	9 (47.4)	
Lateralization	Right	45 (52.3)	20 (51.3)	25 (53.2)	0.516	36 (53.7)	9 (47.4)	0.408
	Left	41 (47.79)	19 (48.7)	22 (46.8)		31 (46.3)	10 (52.6)	
Tumor location	Frontal	22 (25.6)	13 (33.3)	9 (19.1)	0.157	19 (28.4)	3 (15.8)	0.143
	Parietal	18 (20.9)	8 (20.5)	10 (21.3)		14 (20.9)	4 (21.1)	
	Occipital	15 (17.5)	6 (15.4)	9 (19.1)		13 (19.4)	2 (10.5)	
	Temporal	31 (36.0)	12 (30.8)	19 (40.4)		21 (31.3)	10 (52.6)	
Adjuvant chemo	No	38 (44.2)	20 (51.3)	18 (38.3)	0.161	30 (44.8)	8 (42.1)	0.524
	Yes	48 (55.8)	19 (48.7)	29 (61.7)		37 (55.2)	11 (57.9)	
Radiotherapy	No	23 (26.7)	15 (38.5)	8 (17.0)	0.023	19 (28.4)	4 (21.1)	0.376
	Yes	63 (73.3)	24 (61.5)	39 (83.0)		48 (71.6)	15 (78.9)	
Selected chemo regimen	Temozolomide	34 (70.1)	13 (68.4)	21 (72.4)	0.507	25 (67.6)	9 (81.2)	0.305
	Irinotecan plus Bevacizumab	14 (29.9)	6 (31.6)	8 (27.6)		12 (32.4)	2 (18.2)	
Tumor focality	Unifocal	62 (72.1)	24 (61.5)	38 (80.9)	0.040	46 (68.7)	16 (84.2)	0.148
	Multifocal	24 (27.9)	15 (38.5)	9 (19.1)		21 (31.3)	3 (15.8)	
Tumor size, mm	<50	44 (51.2)	23 (59.0)	21 (44.7)	0.135	37 (55.2)	10 (52.6)	0.522
	≥50	42 (48.8)	16 (41.0)	26 (55.3)		30 (44.8)	9 (47.4)	
ATRX loss	None	76 (88.4)	35 (89.7)	41 (87.2)	0.494	36 (53.7)	8 (42.1)	0.263
	Present	10 (11.6)	4 (10.3)	6 (12.8)		31 (46.3)	11 (57.9)	
Ki67	≤%50	59 (68.6)	26 (66.7)	33 (70.2)	0.451	57 (85.1)	19 (100.0)	0.070
	>%50	27 (31.4)	13 (33.3)	14 (29.8)		10 (14.9)	0 (0.0)	
Surgical procedure	Subtotal	47 (54.7)	24 (61.5)	23 (48.9)	0.171	45 (67.2)	14 (73.7)	0.405
	Gross total	39 (45.3)	15 (38.5)	24 (51.1)		22 (32.8)	5 (26.3)	
p53 status	Wild	41 (47.7)	18 (46.2)	23 (48.9)	0.484	33 (49.3)	8 (42.1)	0.387
	Mutant	45 (52.3)	21 (53.8)	24 (51.1)		34 (50.7)	11 (57.9)	

Abbreviations: ECOG PS, Eastern Cooperative Oncology Group Performance Status; ATRX, alpha thalassemia/mental retardation syndrome X-linked; PD-L1, programmed cell death ligand-1.

**Table 2:** Univariate Cox regression analysis of survival in patients with IDH wild-type glioblastoma.

Overall Survival					Progression-Free Survival				
	HR	95.0% CI		*p*		HR	95.0% CI		*p*
		Lower	Upper				Lower	Upper	
Age	2.56	1.50	4.37	0.001	Age	1.86	1.11	3.10	0.018
Sex	0.99	0.59	1.65	0.972	Sex	0.87	0.52	1.45	0.615
Comorbidity	2.07	1.22	3.51	0.007	Comorbidity	1.43	0.86	2.38	0.161
ECOG PS	1.69	1.01	2.81	0.042	ECOG PS	1.48	0.89	2.46	0.127
Tumor location	0.96	0.78	1.19	0.766	Tumor location	0.98	0.79	1.21	0.858
Lateralization	0.98	0.59	1.63	0.949	Lateralization	0.97	0.58	1.61	0.923
Adjuvant chemo	0.74	0.44	1.23	0.254	Adjuvant chemo	0.94	0.56	1.56	0.815
Radiotherapy	0.97	0.54	1.75	0.929	Radiotherapy	1.16	0.64	2.10	0.616
Selected chemo regimen	0.35	0.15	0.79	0.012	Selected chemo regimen	0.44	0.19	1.01	0.054
Tumor focality	1.26	0.71	2.23	0.422	Tumor focality	1.06	0.60	1.86	0.830
Surgical procedure	0.66	0.38	1.11	0.123	Surgical procedure	0.77	0.46	1.28	0.318
Tumor size	1.09	0.66	1.82	0.718	Tumor size	1.04	0.63	1.73	0.858
ATRX loss	1.53	0.75	3.13	0.241	ATRX loss	1.31	0.64	2.68	0.447
Ki67	0.95	0.54	1.66	0.874	Ki67	0.91	0.52	1.58	0.744
p53 status	0.75	0.45	1.25	0.278	p53 status	0.72	0.43	1.20	0.215
B7-H3 expression	1.98	1.16	3.40	0.012	B7-H3 expression	1.95	1.14	3.32	0.014
PD-L1 expression	0.87	0.46	1.66	0.687	PD-L1 expression	0.83	0.44	1.56	0.570

Abbreviations: ECOG PS, Eastern Cooperative Oncology Group Performance Status; ATRX, alpha thalassemia/mental retardation syndrome X-linked; PD-L1, programmed cell death ligand-1.

**Table 3:** Multivariate Cox regression analysis of survival in patients with IDH wild-type glioblastoma.

Overall Survival					Progression-Free Survival				
	HR	95.0% CI		*p*		HR	95.0% CI		*p*
		Lower	Upper				Lower	Upper	
Age	1.86	0.80	4.32	0.147	Age	2.06	1.22	3.48	0.007
Comorbidity	1.29	0.56	2.92	0.542	Comorbidity	-	-	-	-
ECOG PS	1.17	0.50	2.70	0.711	ECOG PS	-	-	-	-
Selected chemo regimen	0.20	0.07	0.56	0.002	Selected chemo regimen	-	-	-	-
B7-H3 expression	6.47	2.58	16.21	<0.001	B7-H3 expression	2.14	1.25	3.67	0.005

Abbreviations: ECOG PS, Eastern Cooperative Oncology Group Performance Status.

## Data Availability

The datasets used in this study can be made available by the corresponding author upon reasonable request, with permission from the Pathology Department of Sivas Cumhuriyet University School of Medicine.
